# Homomorphic Filtering and Phase-Based Matching for Cross-Spectral Cross-Distance Face Recognition

**DOI:** 10.3390/s21134575

**Published:** 2021-07-04

**Authors:** Fitri Arnia, Maulisa Oktiana, Khairun Saddami, Khairul Munadi, Roslidar Roslidar, Biswajeet Pradhan

**Affiliations:** 1 Department of Electrical and Computer Engineering, Universitas Syiah Kuala, Banda Aceh 23111, Indonesia; maulisa@mhs.unsyiah.ac.id (M.O.); khairun.saddami@unsyiah.ac.id (K.S.); khairul.munadi@unsyiah.ac.id (K.M.); roslidar@unsyiah.ac.id (R.R.); 2 Telematics Research Center (TRC), Universitas Syiah Kuala, Banda Aceh 23111, Indonesia; 3 Centre for Advanced Modelling and Geospatial Information Systems (CAMGIS), Faculty of Engineering and IT, University of Technology Sydney, Ultimo, NSW 2007, Australia; Biswajeet.Pradhan@uts.edu.au or; 4 Department of Energy and Mineral Resources Engineering, Sejong University, Choongmu-gwan, 209 Neungdong-ro, Gwangjin-gu, Seoul 05006, Korea; 5 Earth Observation Center, Institute of Climate Change, Universiti Kebangsaan Malaysia, Bangi 43600 UKM, Selangor, Malaysia

**Keywords:** BLPOC, cross-spectral face recognition, cross-distance face recognition, photometric normalization

## Abstract

Facial recognition has a significant application for security, especially in surveillance technologies. In surveillance systems, recognizing faces captured far away from the camera under various lighting conditions, such as in the daytime and nighttime, is a challenging task. A system capable of recognizing face images in both daytime and nighttime and at various distances is called Cross-Spectral Cross Distance (CSCD) face recognition. In this paper, we proposed a phase-based CSCD face recognition approach. We employed Homomorphic filtering as photometric normalization and Band Limited Phase Only Correlation (BLPOC) for image matching. Different from the state-of-the-art methods, we directly utilized the phase component from an image, without the need for a feature extraction process. The experiment was conducted using the Long-Distance Heterogeneous Face Database (LDHF-DB). The proposed method was evaluated in three scenarios: (i) cross-spectral face verification at 1m, (ii) cross-spectral face verification at 60m, and (iii) cross-spectral face verification where the probe images (near-infrared (NIR) face images) were captured at 1m and the gallery data (face images) was captured at 60 m. The proposed CSCD method resulted in the best recognition performance among the CSCD baseline approaches, with an Equal Error Rate (EER) of 5.34% and a Genuine Acceptance Rate (GAR) of 93%.

## 1. Introduction

In biometric systems, face recognition has become the most popular research area [1,2,3,4,5,6,7,8]. One of the factors that contributes to its popularity is the extensive use of surveillance cameras in various applications [9]. In the past two decades, numerous face recognition methods have been developed to recognize a person for various purposes, such as criminal detection, law enforcement, image spoofing, and other security applications [10,11,12,13,14,15,16,17]. The pioneer of face recognition utilizes either the visible light images or infrared images to identify a person [10,11,12]. The recognition of these two types of images is done in the same spectral band. In addition, some efforts to apply deep learning in face image recognition have been demonstrated in [14,15,16,17]. These works also considered the recognition between images in the same spectral band, i.e., the visible images and their various versions.

In recent years, facial images used for the identification purpose are captured at different electromagnetic spectra and wavelengths, namely, Near-Infrared (NIR), Visible Light (VIS), Short Wavelength Spectrum (SWIR), and Long Wavelength Infrared (LWIR) [14]. A face recognition system using different spectra, such as VIS and NIR, referred to as cross-spectral (CS) face recognition, is becoming more attractive because of the benefits it potentially brings in the security area and surveillance applications, such as helping to identify a criminal [18,19,20,21,22]. In the surveillance context, CS face recognition schemes can be useful in extreme situations or harsh environments, where the identification and verification process are done in less controlled environments.

Moreover, the latest development in digital cameras has allowed both VIS and NIR images to be captured at various distances and with good quality. The availability of these images leads to the development of face recognition methods between VIS and NIR at cross-distance, referred to as cross-spectral cross distance (CSCD) face image recognition. Considering that we are encountering more challenging surveillance circumstances, CSCD can further improve the surveillance technology. Some of its potential applications are in the surveillance network for public safety in a smart city, border control, and body-worn cameras for mobile-based surveillance.

Previous CSCD approaches [9,23,24,25] applied a typical face recognition procedure, which consists of three steps: (1) preprocessing, (2) feature extraction, and (3) distance/ threshold calculation. The preprocessing step aims to prepare the image prior to feature extraction. Normally, a normalization filter is applied to tune some parameters such as spatial position and image orientation, to obtain an acceptable recognition performance [26]. Photometric normalization is particularly done to eliminate uneven illumination caused by the nature of image acquisition circumstances. Several photometric normalization methods that can be used to address uneven illumination are Homomorphic filtering, DCT-based filtering, and TanTriggs filtering [27]. The compatibility of a particular feature to a particular domain/application must be taken into account, and the extracted feature must represent image information efficiently. Finally, the distance measure must be selected appropriately to achieve a high recognition rate. Each of these steps depends on each other, and the performance of the previous steps can affect the subsequent ones.

In this article, we proposed a phase-based CSCD face recognition system. We employed Homomorphic filtering as a photometric normalization technique [28], and a phase-based image matching method—known as Band Limited Phase Only Correlation (BLPOC) [29]—in place of feature extraction and recognition steps. Different from the state-of-the-art, the phase component of an image is directly used, without the need of feature extraction. Thus, the effectiveness of the selected feature will not be an issue. To the best of our knowledge, this study is the first in the literature to employ a phase-based correlation for cross-spectral cross-distance (CSCD) face recognition. Moreover, the proposed method outperforms the state-of-the-art approaches, such as those in [9,23,24,25].

The major contributions of this paper are as follows:

1. We introduce the CSCD face recognition based on a Homomorphic filtering and phase-based matching method, which has a higher recognition rate than the state-of-the-art methods in the field.
2. We propose a simpler CSCD face recognition method, which eliminates the effort required to select an appropriate feature and distance measurement.
3. We confirm that the Homomorphic filtering is the most robust filter to distance changing in CSCD framework.

The remainder of this paper is organized as follows. Section 2 briefly reviews the CS and CSCD frameworks, as well as some related works. Section 3 explains our proposed method, while Section 4 describes the experimental setting. Section 5 presents the results and discussion, and Section 6 concludes the study.

## 2. Related Works

**Figure 1:**
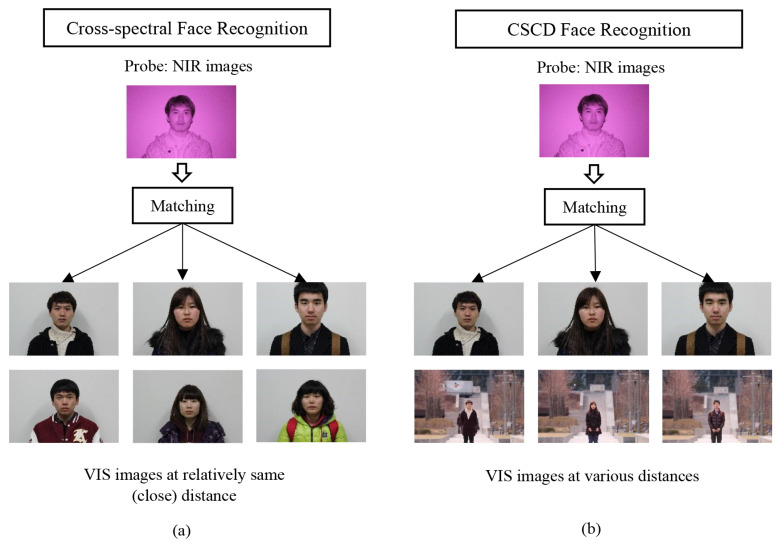
(**a**) Cross-Spectral (CS) and (**b**) Cross-spectral Cross-Distance (CSCD) Face Recognition.

Figure 1 illustrates the face recognition scheme in (a) CS and (b) CSCD frameworks. CS matching refers to the matching of two face images captured under different spectra to provide a more accurate facial description [30,31]. In the CS system, the face images captured under the NIR spectral band are matched with the face images captured under the VIS spectral band. In the VIS spectral band, facial descriptions of people from different races show different characteristics [25]. In contrast, the NIR spectral band utilizes the calibrated IR sensor to overcome the different race factors such as skin color and facial characteristics. Due to this reason, the CS matching scenarios provide a more accurate facial recognition because they utilize a complementary facial description at different wavelengths. The complementary facial description can reveal facial features in a certain spectrum that may not be observable in another spectrum. The main concern in CS is to eliminate uneven illumination of images occurring in both spectra. We refer to CSCD when the probe and gallery images are captured under different spectra and at the different distances. When the images are captured at a long distance, another major issue in the CSCD arises in addition to uneven illumination, namely, deteriorated image quality.

Zuo et al. [32] evaluated the cross-spectral face matching between the face image captured under the spectral band and those captured under the SWIR spectral band. Local Binary Pattern (LBP) and Generalized Local Binary Pattern (GLBP) features were used for the encoding process. An adaptive score normalization method was used to improve the recognition performance. The approach resulted in a better recognition performance. However, the improved performance highly depends on a score level fusion scenario.

Klare and Jain [33] performed the cross-spectral face matching between NIR and VIS face images using the Local Binary Pattern (LBP) and Histogram of Oriented Gradient (HoG) features. The encoding process relied on the Linear Discriminant Analysis (LDA). Moreover, the previous methods [32,33] explored the cross-spectral face matching with relatively similar (close) distances, where the probe images and gallery data used for matching were captured at the same distance (either short-range or long-range).

Kalka et al. [23] pioneered the work in cross-spectral face image matching under various scenarios. The face images were captured under various conditions and scenarios, such as at a close distance, steady standoff distance (2 m), with frontal faces and facial expressions, and in indoors and outdoors. Kalka used the VIS spectral band as a gallery data and the SWIR spectral band (at 1550 nm) as a probe image.

In 2013, Maeng et al. [24] explored the Gaussian filtering and Scale Invariant Feature Transform (SIFT) for CSCD face matching. The noise at high frequency was reduced by using the Gaussian filtering. The facial features were then extracted using the SIFT feature extraction method.

Kang et al. [9] proposed the CSCD method, in which the Heterogeneous Face Recognition system (HFR) was employed. In the study, the HFR algorithm utilized three kinds of filters and two kinds of descriptors for the encoding process. The filters used in the HFR algorithm were Difference of Gaussian (DoG), Center-Surround Divisive Normalization (CSDN), and Gaussian, while the descriptors used were Scale Invariant Feature Transform (SIFT) and Multiscale Local Binary Pattern (MLBP). The facial representation was achieved by combining all of the features from the overlapped patches.

Shamia and Chandy [25] examined the use of a combination of wavelet transform, Histogram-Oriented Gradient (HOG), and Local Binary Pattern (LBP) for CSCD face matching. In their study, the VIS spectral band was used as a gallery image, while the NIR spectral band was used as a probe image. To reduce the gap between the NIR and VIS images, the VIS image’s contrast was enhanced using the Difference of Gaussian filtering (DoG), while the NIR image’s contrast was enhanced using median filtering. Note that the earlier works require a three-step recognition procedure: preprocessing, feature extraction, and distance calculation.

To the best of our knowledge, CSCD face recognition has not been addressed by a deep learning technique. The work proposed by Pini et al. [34] used deep learning for cross-distance and cross-device face recognition. The cross-distance experiments were carried out using the same device, while the cross-device tests were run at a fixed distance. The aim was to identify the best combination of data representation, preprocessing/normalization technique, and deep learning model that obtain the highest recognition accuracy rate. In addition, Pini proposed an image dataset named MultiSFace, which contains visual (VIS) and infrared images, high- and low- resolution depth images, and high- and low- resolution thermal images, captured from two different distances: near (1 m) and far (2.5 m). Instead of presenting the recognition results between the images of different spectra (VIS and infrared), the work only discussed the recognition results between several depth map representations of face, namely, normal images, point clouds, and voxels, generated by different devices. Note that normal images, point clouds, and voxels are all the derivation of the VIS images.

## 3. Proposed Method

**Figure 2:**
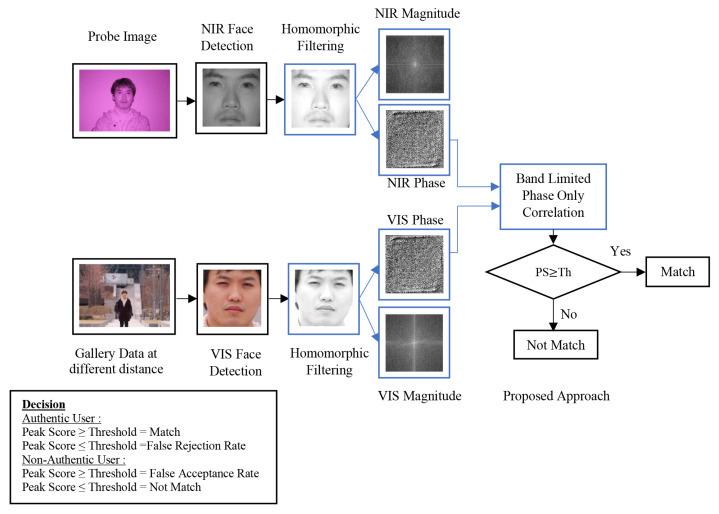
The proposed framework.

Figure 2 illustrates the overview of the proposed approach. Here, the NIR images captured at a short distance, were used as a probe image, while the VIS images captured at a longer distance, were used as gallery image. Each block in the diagram is explained as follows:

### 3.1. Face Detection

The Viola Jones face detection [35] was used to detect the facial area. The Viola and Jones face detection is computationally simpler than the recent Convolutional Network approaches, such as Multi-Task Cascaded Convolutional Networks (MTCNN) [36], because it does not require expensive annotation as the CNN (MTCNN) does for image training.

There are two steps in the Viola Jones face detection system: training and detection. The detection step consists of two sub-steps: selecting the Haar features and creating an integral image. The training step also has two sub-steps: training the classifiers and applying the Adaboost. Here, the Viola Jones face detection steps are implemented as follows [37]:

1. Convert the NIR and VIS images to grayscale, as the Viola–Jones algorithm detects the facial area within the grayscale image and searches the corresponding location on the colored image.
2. Divide the NIR and VIS images into block windows. Every block is scanned from the left to the right.
3. Compute the facial feature using Haar-like features of each block. The Haar feature can be obtained by subtracting the pixel in the black area with the pixel in the white area.
4. Convert the input image into an integral image. Then, apply Adaboost to select features and train the feature by cascading process.
5. Concatenate all the Haar-like features in each block window to determine the location of the facial area.

### 3.2. Homomorphic Filtering

The Homomorphic filtering technique aims to reduce the illumination variation as a result of different lighting conditions [38,39,40]. The illumination variations can be normalized using the filter function. The filter was set to decrease the effects of the illumination (in the low-frequency components primarily) and amplifies the reflectance (in the most of the high-frequency components). Previous work has shown that Homomorphic filtering is suitable to reduce the cross-spectral appearance differences [27]. Therefore, in this paper, homomorphic filtering was used to address the modality issue between two images captured at different spectral bands.

After the face detection stage, homomorphic filtering is applied to the NIR and VIS face images to enhance the facial features. Both the NIR and VIS face images are processed through similar steps. For simplicity, the NIR and VIS face images are annotated as I(x,y). The following are the Homomorphic filtering steps applied to both NIR and VIS images [27]:

1. The face images I(x,y) are transformed into the logarithmic form.
  (1) Z(x,y)=log(I(x,y))
2. The logarithmic images (Z(x,y)) are then transformed into a frequency domain using the Fourier transform.
  (2) F{Z(x,y)}=F{log(I(x,y))}
  For simplicity:
  (3) $$Z\left(u,v\right)=F_{I}\left(u,v\right)$$
  Here, Z(u,v) represents the image in the frequency domain, while $F_{I}\left(u,v\right)$ represents the Fourier transform of log(I(x,y)).
3. Then, the images are multiplied with a high-pass filter H(u,v), which corresponds to a convolution operation in the spatial domain.
  (4) C(u,v)=Z(u,v)×H(u,v)
  Here, C(u,v) denotes the filtered image in the frequency domain.
4. The filtered images in the spatial domain C(x,y) are obtained by taking the inverse Fourier transform of Equation (4).
  (5) $$C\left(x,y\right)=F^{-1}\left\{C\left(u,v\right)\right\}$$
5. The Homomorphic image $h_{0}\left(x,y\right)$ can be obtained by taking the exponential of C(x,y).
  (6) $$h_{0}\left(x,y\right)=exp\left\{C\left(x,y\right)\right\}$$

### 3.3. Band Limited Phase Only Correlation

After the Homomorphic filtering step, the resulted Homomorphic of VIS and NIR images were then transformed using the discrete Fourier transform (DFT) as follows:

(7) $$\begin{array}{c} VIS\left(n_{1},n_{2}\right)\Rightarrow VIS\left(k_{1},k_{2}\right)=A_{VIS}\left(k_{1},k_{2}\right)e^{j\theta_{VIS}\left(k_{1},k_{2}\right)}\\ NIR\left(n_{1},n_{2}\right)\Rightarrow NIR\left(k_{1},k_{2}\right)=A_{NIR}\left(k_{1},k_{2}\right)e^{j\theta_{NIR}\left(k_{1},k_{2}\right)} \end{array}$$

$VIS(n_{1},n_{2})$ and $NIR(n_{1},n_{2})$ are VIS and NIR images in the spatial domain while $VIS(k_{1},k_{2})$, $NIR(k_{1},k_{2})$ are 2D DFT of the VIS and NIR images. $A_{VIS}\left(k_{1},k_{2}\right)$, $A_{NIR}\left(k_{1},k_{2}\right)$ represents the amplitude component of VIS and NIR images respectively. $\theta_{VIS}\left(k_{1},k_{2}\right)$ is the phase component of the VIS images while $\theta_{NIR}\left(k_{1},k_{2}\right)$ is the phase component of the NIR images. The normalized cross-power spectrum is then used to compute the phase differences between the VIS and NIR images as described in [28].

(8) $$R_{VISNIR}\left(k_{1},k_{2}\right)=\frac{VIS\left(k_{1},k_{2}\right)\bar{NIR(k_{1},k_{2})}}{|VIS\left(k_{1},k_{2}\right)\bar{NIR(k_{1},k_{2})}|}$$

Here, $\bar{NIR(k_{1},k_{2})}$ is the complex conjugation of the NIR images. $R_{VISNIR}\left(k_{1},k_{2}\right)$ represents the normalized cross-power spectrum between the VIS and NIR images. The frequency band is set only to keep the most important phase spectrum information. Therefore, Band-limited Phase-Only Correlation (BLPOC) can result in a maximum correlation peak between the two images. If the two images are similar, the BLPOC will result in a maximum correlation peak score. If the two images are different, the BLPOC will result in a minimum correlation peak score.

A threshold is determined to assess the peak score values. Based on the assessment, a decision is made. The decision rules are as follows:

- For authentic users (probe image is member of gallery data).
  1. If the peak score > threshold, the probe matches the gallery image; then, the probe is verified/recognized.
  2. If the peak score < threshold, it is a false rejection rate.
- For non-Authentic User (probe image is a non member of gallery data).
  1. If the peak score > threshold, it is a false acceptance rate.
  2. If the peak score < threshold, the probe does not match the gallery image; consequently the probe is not verified/not recognized.

## 4. Experimental Setting

**Figure 3:**
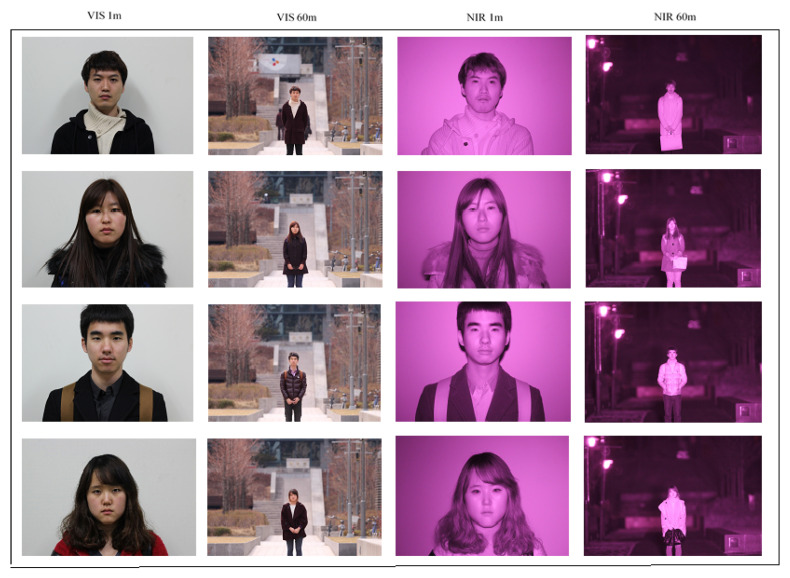
The sample of NIR and VIS face images captured at 1m and 60 m distance.

The experiment was conducted using the Long-Distance Heterogeneous Face database (LDHF-DB) [9]. The whole database was collected over a period of 1 month at the Korea University, Seoul. The LDHF-DB database consisted of both frontal VIS and frontal NIR face images of 100 different subjects (70 males and 30 females), captured at 1 m, 60 m, 100 m, and 150 m standoff in an outdoor environment. The resolution of the images was 5184 × 3456 pixels and the images were stored in both JPEG and RAW formats. Figure 3 shows the sample of cross spectral face images captured at 1 m and 60 m.

We examined two main aspects of the proposed method: (a) the effects of BLPOC frequency band (BLPOC FB) variation on filtering operations and (b) the performances of filtering operations schemes at the best BLPOC FB obtained in (a). From each aspect, we evaluated the simulation results in three scenarios:

- (i) CS face verification at 1m (short distance),
- (ii) CS face verification at 60 m (long distance), and
- (iii) CSCD face verification where the probe images (NIR face images) were captured at 1 m while the gallery data (VIS face images) were captured at 60 m.

Finally, (c) we compared the performance the proposed method with the existing CSCD baseline methods [9,24,25].

In each scenario, we also applied other photometric normalization filters, namely, TanTriggs filter and DCT filter, for comparison purposes. These filters were employed to filter the NIR and VIS images, replacing the Homomorphic filters (see Figure 2). We also evaluated a condition in which the face detection step is directly followed by BLPOC. We refer to this condition as “No-filter”.

The experiment was performed using 100 NIR images as the probe images and 100 VIS images as the gallery images for both 1 m and 60 m distance. The total number of matching comparisons was 10,000 for each distance, while the total number of the genuine comparisons was 100, and that of impostor comparisons was 9900.

The recognition performance was evaluated using the Equal Error Rate (EER) and Receiver Operating Characteristic (ROC) curve parameters. The EER parameter is a single value, in which the False Acceptance Rate (FAR) is equal to the False Rejection Rate (FRR) while the ROC curves compute the ratio between the recognition rate (Genuine Acceptance Rate (GAR)) and FAR at different reference thresholds. The reference thresholds were calculated as in [28].

## 5. Results and Discussion

### 5.1. The Effect of Blpoc fb Variation

**Figure 4:**
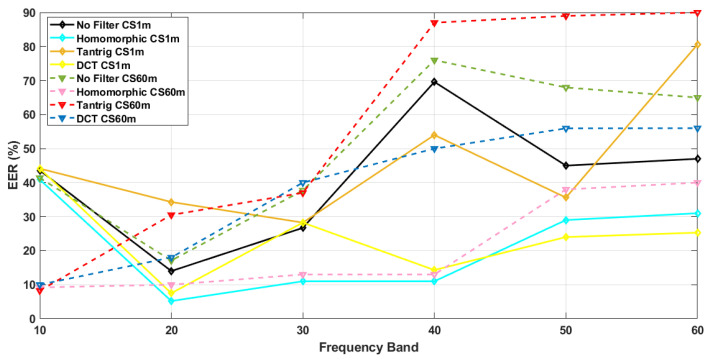
The effects of BLPOC FB variations on filtering operations in CS face recognition.

**Figure 5:**
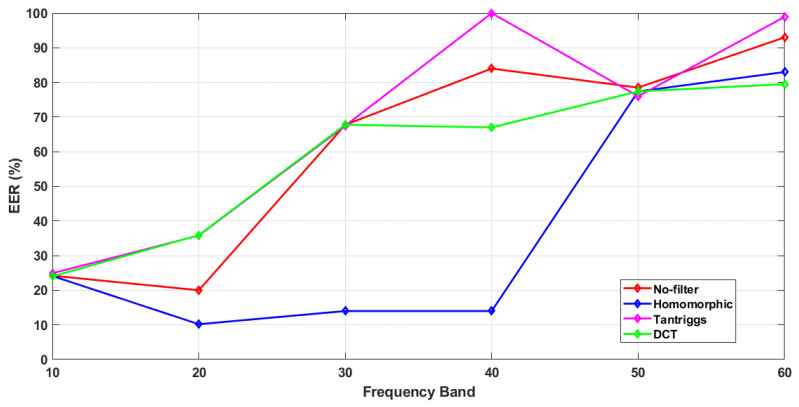
The effects of BLPOC FB variations on filtering operations in CSCD face recognition.

Table 1 presents the EER values and recognition rates of the proposed method and all comparison methods, which were calculated at six different ranges of BLPOC FBs, i.e., 10, 20, 30, 40, 50, and 60. From Table 1, we extracted the EER values of CS and CSCD, and plotted them as a function of BLPOC FB variations. Figure 4 and Figure 5 show the effects of BLPOC FB variation on filtering operations in both CS (scenario (i) and (ii)) and CSCD (scenario (iii)) face recognition, respectively.

#### 5.1.1. CS Face Recognition

In Figure 4, CS face verification at 1 m (scenario (i)) is plotted by solid lines, and that at 60 m (scenario (ii)) is plotted by dashed lines. Roughly, in both scenarios, the combination of BLPOC and Homomorphic filter resulted in the smallest EER values (i.e., the best performances) as the frequency band increased (see solid blue line and dashed pink line).

In scenario (i), the EER values of the proposed method shows a steady increment as BLPOC FB increased. Here, the EER values were 40.9%, 5.2%, 11%, 11%, 29%, and 31% as the frequency band increased from 10 to 60. The EER value at the frequency band 20 declined, but then the EER values increased steadily. Thus we consider frequency band 20 as the breakdown point, where the method resulted in the smallest EER value. In scenario (ii), the EER values of the proposed method were 9.2%, 10%, 13%, 13%, 38%, and 40% as the frequency band increased. Here, the EER values were steadily increased from frequency band 10 to 60, and there was no breakdown point.

On the other hand, the EER values of the comparison methods showed either some irregular fluctuations, or no particular breakdown point as the BLPOC FB increased. For example, the EER values of the combination of BLPOC and TanTriggs filter (see solid dark yellow line) in scenario (i) were 44.14%, 34.3%, 28.5%, 54%, 35.6%, and 80.6%. The EER values of BLPOC combined with DCT filter (see solid bright yellow line) in scenario (i) also fluctuated. In scenario (ii), the EER values of BLPOC combined with both TanTriggs and DCT filters increased steadily as the BLPOC FB increased. In these cases, there were no breakdown points.

#### 5.1.2. CSCD Face Recognition

As shown in Figure 5, the proposed method (see the blue line) resulted in the smallest EER values with the increment of BLPOC FB (except at the frequency band 50 and 60). The breakdown point of the EER values was at the frequency band 20.

At the frequency band 10, the EER value was 24.12%. The EER value decreased to 10.2% at the frequency band 20, and the value continued to increase at the subsequent frequency bands. At the breakdown point, the proposed method resulted in 10.2% EER and recognition rate of 93%, which is the best trade-off between EER value and recognition rate (see Table 1).

On the contrary, the EER values of other comparison methods have either more fluctuations or higher breakdown point at a larger BLPOC FBs. For example, the EER values of combination of No-filter and BLPOC have breakdown points at the frequency band 20 and 50. The EER values of DCT filter combined with BLPOC broke down slightly at the frequency band 40 (the EER at the frequency band 30 was 67.78%, which declined to 67% at the frequency band 40, and increased to 77.4% at the frequency band 50). The breakdown point of TanTriggs filter combined with BLPOC was at the frequency band 50, with 76% EER. Wherever the breakdown points are, the EER values of the comparison methods were far greater than those of the proposed method, which means that the performances of the comparison methods were poorer than that of the proposed method.

In Table 1, an anomaly is observed in the EER values of BLPOC-FB 10. Primary assumptions of EER values in each FB are that the EER value of scenario (i) should be the smallest, the EER values of scenario (ii) should be higher than those of scenario (i), but lower than those of scenario (iii). In other words, the image recognition at 1m should be easier than that at 60m, and image recognition in the CS scenario should be easier than that in the CSCD scenario. However, at the frequency band 10, most of the EER values of scenario (ii) were lower than those of scenario (i) and scenario (iii). In our proposed method, the filtering operation was followed by BLPOC. The filters reduced the illumination (low-frequency) part and enhanced the reflectance (high-frequency) part, which contained the detail of the images. Then, the BLPOC-FB 10 limited these frequencies to only 10 lowest frequencies, excluding the enhanced reflectance component. This exclusion may result in the anomaly.

As shown in this section, the combination of BLPOC and Homomorphic filter at the frequency band 20 had the best trade-off between EER value and recognition rate (scenario (i) and (iii)). In scenario (ii), the proposed method achieved the best performance (the smallest EER) at BLPOC-FB 10, with 9.2% EER. Furthermore, even at BLPOC FB 20, the proposed method had the smallest EER compared to those of the comparison methods. Thus, in the following, we suggest that a further investigation on performance of photometric normalization schemes evaluated at BLPOC FB 20, is necessary.

### 5.2. Performance of Photometric Normalization

**Figure 6:**
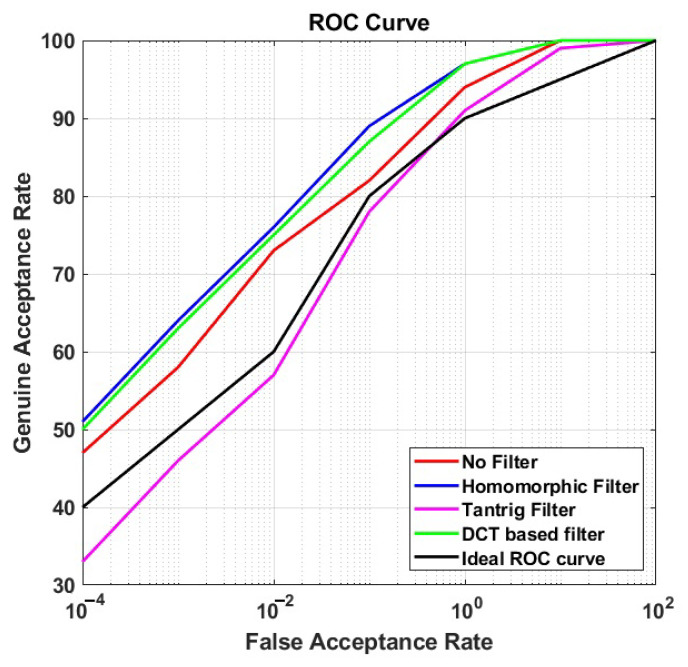
The ROC curves of the proposed method applied to CS face matching at a short distance (1 m) with the BLPOC frequency band 20.

**Figure 7:**
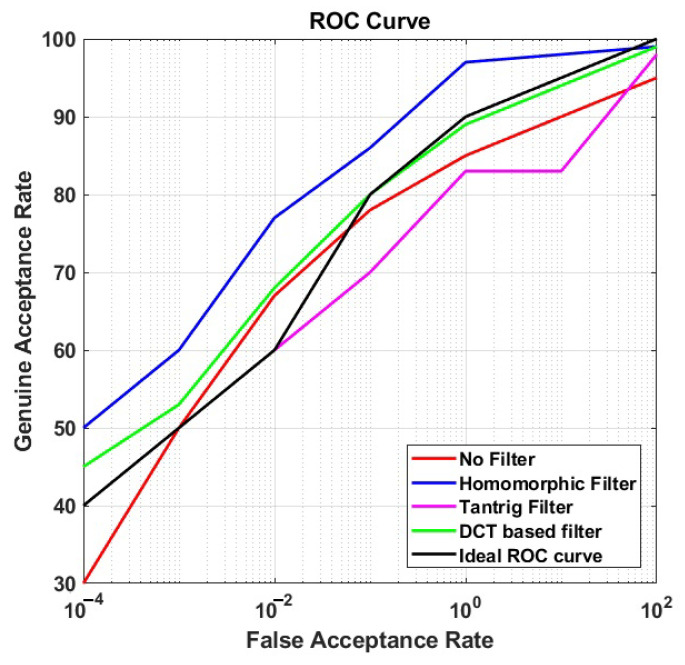
The ROC curves of the proposed method applied to CS face matching at a long distance (60 m) with the BLPOC frequency band 20.

**Figure 8:**
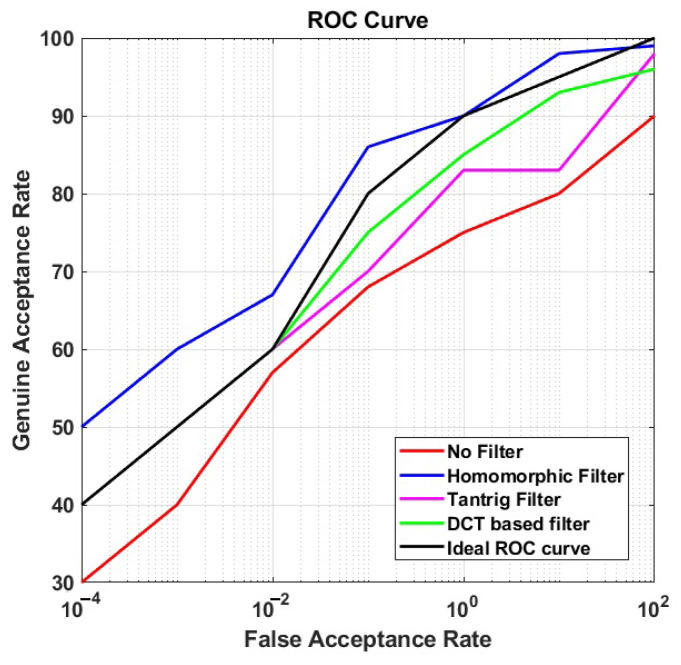
The ROC curves of the proposed method in CSCD face matching.

In this section, we present the performance (ROC curves) of each photometric normalization filter at BLPOC FB 20 in CS and CSCD face recognition. We plotted those ROC curves in Figure 6 and Figure 7 for CS face recognition (scenario (i) and (ii)), and Figure 8 for CSCD face recognition (scenario (iii)). We also present the ideal ROC curve calculated according to the work in [1] to further analyze the performance of the proposed method.

#### 5.2.1. CS Face Recognition

As shown in Figure 6 and Figure 7, the proposed method achieved the highest performance in both short- and long-distance face recognition, compared to other comparison methods, with 97% GAR at 1% FAR, and 98% GAR at 1% FAR, respectively. Moreover, the GAR values of face recognition at the long distance were steady, and the ROC curves remained above the ideal ROC curve.

On the other hand, the GAR values of the comparison methods reduced, and the corresponding ROC curves moved closer to the ideal ROC curve when the image recognition was conducted at a long distance. For instance, in scenario (i), the overall ROC curve of DCT filter was positioned at the second rank (with 97% GAR at 1% FAR—the same value as the proposed method). However, in scenario (ii), though ROC curve of DCT filter remained at the second rank, the GAR values at 1% FAR reduced to less than 90%. Moreover, in both scenarios, the ROC curve of TanTriggs filter was mostly positioned under that of No-filter. It implies that the recognition performance without using any filters was better than that using the TanTriggs filter. When the ideal ROC curve was used as a benchmark, it is observable that the proposed method held on its best performance, while those of the comparison methods declined, when the recognition is performed at a long distance. It indicates that the proposed method is more robust to long-distance CS face recognition.

#### 5.2.2. CSCD Face Recognition

Figure 8 shows that in CSCD face recognition, the ROC of the proposed method continued to position above the ideal ROC curve, with 90% GAR at 1% FAR. On the contrary, the ROC values of all other photometric normalization filters moved down under the ideal ROC curve. In this case, it is demonstrated that combination of Homomorphic filtering and BLPOC is effective in cross-spectral cross-distance matching scenarios.

Table 2 summarizes the matching performance using Homomorphic filtering in CSCD matching scenarios. Homomorphic filtering provides a better recognition performance in CS and CSCD scenarios with 5.2% EER at 97% GAR at 1m stand-off, 5.25% EER at 94% GAR at 60m stand-off, and 5.34% EER at 93% GAR, respectively. The recognition rates in CS scenario slightly decreased as the distance become longer. Furthermore, the recognition rate of CSCD decreased compared to those of CS, but only in a small proportion. Overall, the proposed method has shown a steady performance for CS, and it is robust for CSCD framework.

### 5.3. Comparison with Baseline Cscd and Other Methods

Table 3 presents the comparison of the proposed method with the baseline CSCD face recognition methods [9,24,25]. All baseline methods used the Long-Distance Heterogeneous Face Database (LDHF-DB). The method of Kang et. al. resulted in 73.7% GAR at 1% FAR and an EER of 8.6%. The GAR of Maeng’s method was 81% at 1% FAR, while the method of Shamia and Chandy resulted in 72% GAR at 1%FAR. The proposed CSCD method, which integrates Homomorphic filtering and BLPOC, outperformed the baseline approaches with 93% GAR at 1% FAR. The proposed method resulted in an EER of 5.34%.

As mentioned earlier, the baseline methods follow a three-step recognition procedure: preprocessing, feature extraction, and threshold/distance calculation to determine if a face can be recognized/verified. In the preprocessing stage, a normalized image was obtained by employing histogram equalization and smoothing operation [24], while photometric normalization was applied in [9] by applying Different of Gaussian (DoG) and Centre Surround Divisive Normalization (CSDN), and wavelet and DoG [25]. Maeng extracted Scale Invariant Feature Transform (SIFT) features and determined a threshold to Euclidean distance values as a base for verification [24]. Kang et. al. used SIFT and Multi-Scale Local Binary Pattern (MLBP) as features and a threshold in a cosine-based similarity measure for verification [9]. Meanwhile, Shamia and Chandy applied histogram of gradient (HoG) and local binary pattern (LBP) and Euclidean distance [25].

The state-of-the-art methods incorporating the three-step recognition procedure have shown insufficient performance. However, we have a hypothesis that Homomorphic filtering can increase the recognition performance of face recognition in CSCD frameworks. Thus, we conducted an additional study by integrating the Homomorphic filtering with Local Binary Pattern (LBP) features and Hamming distance. This approach resulted in 89.23% GAR at 1% FAR and an EER of 6.9%, which is better than those of the baseline methods. These results confirmed our hypothesis that Homomorphic filter as a means of photometric normalization is more suitable for cross-spectral cross-distance face matching than other methods, such as DoG, and a combination of wavelet and DoG.

The proposed method combined the Homomorphic filter as a means of photometric normalization and BLPOC as a means of matching/recognition. Our experiment proved that this combination can achieve the best performance for CSCD face recognition. In feature-based methods, the selection and representation of the most appropriate features are difficult. On the other hand, the BLPOC method does not depend on feature representation. All information from the phase component of an image is directly used in generating the BLPOC correlation peak the two images.

In addition to feature- and phase-based methods, there has been an effort of using a deep learning approach to solve face recognition challenge in different modalities [34]. However, the work in [34] employed the deep learning methods for cross-modalities environment that are different from those of the CSCD. It uses deep learning for either cross-device or cross-distance face recognition. The cross-distance experiments are conducted with the same device, while the cross-device tests are run at the same distance. We did not find any results related to the infrared images in the study. Instead, the study showed the recognition accuracy rate when the CNNs were tested using image depths, point clouds, and voxels, which derived from visible light domain. Thus, despite the work in [34] using several devices, the recognition was done in the same spectrum, namely, the VIS spectrum. The reported highest accuracy rates of either cross-device or cross-distance were lower than 20%.

The simulation results confirmed that the Homomorphic filtering can reduce the illumination variation between images generated in VIS and NIR spectrum, and at the same time increase the images’ detail in both spectra. Thus, the Homomorphic filtering can produce images with fewer variances between the two spectra and enrich the images’ content. We argue that if these images are fed to a deep network, more distinctive features can be learned by the network, and eventually may increase performance of the CSCD recognition based on a deep learning approach.

## 6. Conclusions

In this research, a cross-spectral cross-distance (CSCD) face recognition method using Homomorphic filtering and phase-based matching is proposed. Homomorphic filtering is able to produce photometric normalized images in cross-spectral dimension: visual (VIS) spectrum and near-infrared (NIR) spectrum. Band-Limited Phase-Only Correlation (BLPOC) method is applied as a means of phase matching. The proposed CSCD method outperforms some standard approaches when evaluated in the short-distance and long-distance cross-spectral face matching. It also outperforms some baseline methods in the CSCD framework. Homomorphic filtering is able to suppress uneven illumination in cross-spectral images. Therefore, the BLPOC can improved the recognition of the cross-spectral faces at various distances. The proposed CSCD method resulted in the highest GAR of 93% at 1% FAR, with an EER of 5.34%. Applying deep learning approaches to further enhance the CSCD face recognition performance will be our future work.

## Figures and Tables

**Table 1:** Effects of BLPOC FB variation on filtering operation in CS and CSCD face recognition.

**F = 10**								
	EER (%)				Recognition Rate/GAR (%)			
**Scenario**	**No-Filter**	**Proposed**	**TanTriggs**	**DCT**	**No-Filter**	**Proposed**	**TanTriggs**	**DCT**
Probe & gallery 1 m (CS)	43.6	40.9	44.14	44.14	99	100	99	99
Probe & gallery 60 m (CS)	41.4	9.2	8.25	10	100	100	100	100
Probe 1 m; gallery 60 m (CSCD)	24.2	**24.12**	24.9	24	100	100	98	100
**F = 20**								
	EER (%)				Recognition Rate/GAR (%)			
**Scenario**	**No-Filter**	**Proposed**	**TanTriggs**	**DCT**	**No-Filter**	**Proposed**	**TanTriggs**	**DCT**
Probe & gallery 1 m (CS)	14	5.2	34.3	7.5	94	97	91	97
Probe & gallery 60 m (CS)	17.1	10	30.5	18	97	94	83	94
Probe 1 m; gallery 60 m (CSCD)	20	**10.2**	35.8	35.9	93	93	88	97
**F = 30**								
	EER (%)				Recognition Rate/GAR (%)			
**Scenario**	**No-Filter**	**Proposed**	**TanTriggs**	**DCT**	**No-Filter**	**Proposed**	**TanTriggs**	**DCT**
Probe & gallery 1 m (CS)	26.7	11	28.25	28.25	98	98	97	98
Probe & gallery 60 m (CS)	37.7	13	37	40	86	87	86	86
Probe 1 m; gallery 60 m (CSCD)	67.75	**14**	67.5	67.78	89	89	89	89
**F = 40**								
	EER (%)				Recognition Rate/GAR (%)			
**Scenario**	**No-Filter**	**Proposed**	**TanTriggs**	**DCT**	**No-Filter**	**Proposed**	**TanTriggs**	**DCT**
Probe & gallery 1 m (CS)	69.6	11	54	14.3	44	96	72	99
Probe & gallery 60 m (CS)	76	13	87	50	35	80	55	75
Probe 1 m; gallery 60 m (CSCD)	84	**14**	100	67	37	73	50	54
**F = 50**								
	EER (%)				Recognition Rate/GAR (%)			
**Scenario**	**No-Filter**	**Proposed**	**TanTriggs**	**DCT**	**No-Filter**	**Proposed**	**TanTriggs**	**DCT**
Probe & gallery 1 m (CS)	45	29	35.6	24	50	60	50	50
Probe & gallery 60 m (CS)	67.97	38	89	55.95	11	22	8	9
Probe 1 m; gallery 60 m (CSCD)	78.5	**77.4**	76	77.4	3	12	11	12
**F = 60**								
	EER (%)				Recognition Rate/GAR (%)			
**Scenario**	**No-Filter**	**Proposed**	**TanTriggs**	**DCT**	**No-Filter**	**Proposed**	**TanTriggs**	**DCT**
Probe & gallery 1 m (CS)	47	31	80.6	25.3	48	57	17	57
Probe & gallery 60 m (CS)	65	40	90	56	10	20	4	13
Probe 1 m; gallery 60 m (CSCD)	93	**83**	98.9	79.5	2	10	0	10

**Table 2:** The performances of CS and CSCD matching using homomorphic filtering.

Scenario	EER (%)	GAR (%)
Probe 1 m; gallery 1 m (CS)	5.2	97
Probe 60 m; gallery 60 m (CS)	5.25	94
Probe 1 m; gallery 60 m (CSCD)	5.34	93

**Table 3:** The performance comparison of the proposed CSCD Method with the baseline methods on LDHF database.

Method	EER (%)	GAR (%)
Kang [9]	8.6	73.7
Maeng [24]	not available	81
Shamia and Chandy [25]	not available	72
Homomorphic and Feature-based	6.9	89.23
Proposed (Homomorphic and BLPOC)	5.34	93

## Data Availability

The study did not report any data.
